# The Highly Divergent Mitochondrial Genomes Indicate That the Booklouse, *Liposcelis bostrychophila* (Psocoptera: Liposcelididae) Is a Cryptic Species

**DOI:** 10.1534/g3.117.300410

**Published:** 2018-01-19

**Authors:** Shiqian Feng, Qianqian Yang, Hu Li, Fan Song, Václav Stejskal, George P. Opit, Wanzhi Cai, Zhihong Li, Renfu Shao

**Affiliations:** * Department of Entomology, College of Plant Protection, China Agricultural University, Beijing 100193, China; † Crop Research Institute, 161 06 Prague 6, Czech Republic; ‡ Department of Entomology and Plant Pathology, Oklahoma State University, Stillwater, Oklahoma 74078; § GeneCology Research Centre, Centre for Animal Health Innovation, School of Science and Engineering, University of the Sunshine Coast, Maroochydore DC, Queensland 4556, Australia

**Keywords:** mitochondrial genome, *Liposcelis bostrychophila*, intraspecific variation, cryptic species, evolution

## Abstract

The booklouse, *Liposcelis bostrychophila* is an important storage pest worldwide. The mitochondrial (mt) genome of an asexual strain (Beibei, China) of the *L. bostrychophila* comprises two chromosomes; each chromosome contains approximate half of the 37 genes typically found in bilateral animals. The mt genomes of two sexual strains of *L. bostrychophila*, however, comprise five and seven chromosomes, respectively; each chromosome contains one to six genes. To understand mt genome evolution in *L. bostrychophila*, and whether *L. bostrychophila* is a cryptic species, we sequenced the mt genomes of six strains of asexual *L. bostrychophila* collected from different locations in China, Croatia, and the United States. The mt genomes of all six asexual strains of *L. bostrychophila* have two chromosomes. Phylogenetic analysis of mt genome sequences divided nine strains of *L. bostrychophila* into four groups. Each group has a distinct mt genome organization and substantial sequence divergence (48.7–87.4%) from other groups. Furthermore, the seven asexual strains of *L. bostrychophila*, including the published Beibei strain, are more closely related to two other species of booklice, *L. paeta* and *L. sculptilimacula*, than to the sexual strains of *L. bostrychophila*. Our results revealed highly divergent mt genomes in the booklouse, *L. bostrychophila*, and indicate that *L. bostrychophila* is a cryptic species.

The mitochondrial (mt) genomes of bilateral animals are usually a single circular chromosome, 15–18 kb in size, and contain 37 genes: two ribosomal RNA (rRNA) genes, 13 protein-coding genes (PCGs), and 22 transfer RNA (tRNA) genes (Wolstenholme 1992; Boore 1999; Lavrov 2007). However, there are two major exceptions: linear mt genomes and multipartite mt genomes. Linear mitochondrial chromosomes have been found in 26 species of cnidarians (Warrior and Gall 1985; Kayal *et al.* 2012, 2016; Smith *et al.* 2012; Zou *et al.* 2012). Multipartite mt genomes have been found in nematodes (Armstrong *et al.* 2000; Phillips *et al.* 2016), thrips (Dickey *et al.* 2015), booklice of the genus *Liposcelis* (Wei *et al.* 2012; Chen *et al.* 2014), and parasitic lice (Shao *et al.* 2012; Jiang *et al.* 2013; Dong *et al.* 2014; Song *et al.* 2014; Herd *et al.* 2015). In two extreme cases, the mt genome of the human body louse, *Pediculus humanus*, has fragmented into 20 circular minichromosomes (Shao *et al.* 2009), and the mt genome of cubozoa has fragmented into eight linear minichromosomes (Kayal *et al.* 2012).

The booklouse, *Liposcelis bostrychophila*, is an important storage pest worldwide (Nayak *et al.* 2014), and has two reproductive modes: parthenogenesis and sexual reproduction; each *L. bostrychophila* strain possesses one of these reproductive modes (Goss 1954; Mockford and Krushelnycky 2008). Multipartite mt genomes have been found in both asexual and sexual *L. bostrychophila* strains. An asexual, parthenogenetic strain of *L. bostrychophila* from Beibei, China (hereafter, BB strain), has two mt chromosomes, 8530 and 7933 bp in size, respectively; each chromosome contains approximate half of the 37 genes typically seen in bilateral animals (Wei *et al.* 2012). Two sexual strains of *L. bostrychophila*, from Arizona in the United States (US), however, differ from the BB strain and from each other in mt genome organizations (Perlman *et al.* 2015). One of the sexual strains, the distorter (hereafter, sexual d) strain, has five minichromosomes, whereas the other sexual strain, the normal strain (hereafter, sexual n), has seven minichromosomes. These minichromosomes are circular, 1276–5626 bp in size; each minichromosome contains one to six genes. Furthermore, the two sexual strains of *L. bostrychophila* also differ substantially from each other in mt gene sequence and gene order. Apparently, there is substantial difference between the sexual and the asexual strains of *L. bostrychophila* in mt genome organization. Previous studies also demonstrated high variation among the asexual strains of *L. bostrychophila* in the mitochondrial 16S sequence (Li *et al.* 2011) and inter simple sequence repeat (ISSR) markers (Wang *et al.* 2016). The unusually high variation observed in *L. bostrychophila* in mt genome organization and gene sequence prompted the question whether or not *L. bostrychophila* is a cryptic species.

A species is considered to be “cryptic” if it contains two or more species that were morphologically indistinguishable and are classified as a nominal species (Beheregaray and Caccone 2007). Cryptic species can be a result of recent speciation in which diagnosable morphological traits have not evolved (Sáez and Lozano 2005). As effective biological control, diagnosis and prevention of pests require accurate species identification, cryptic species can be an issue in this regard (Bickford *et al.* 2007). The mt genome is a rich resource for investigating cryptic species and phylogenetic relationships (Blouin 2002; Shao and Barker 2007), and has been used to investigate cryptic species in insects (Simmons and Scheffer 2004; Williams *et al.* 2006; Torricelli *et al.* 2010; Jia *et al.* 2012; Burger *et al.* 2014; Fučíková and Lahr 2015).

To understand the evolution of the mt genome in *L. bostrychophila*, and whether it is a cryptic species, we sequenced the mt genomes of six more asexual strains of *L. bostrychophila* from China, Croatia and the US.

## Materials and Methods

### Sample collection, identification, DNA extraction, PCR amplification, and sequencing

Six asexual strains of *L. bostrychophila* were collected in grain storage facilities. These strains are “BJ” from Beijing (China), “XSG” from Xinshagang, Guangdong Province (China), “HLM” from Huangliangmeng, Hebei Province (China), “SY” from Sanya, Hainan Province (China), “KA” from Kansas (US), and “CA” from Croatia. All samples were identified as *L. bostrychophila* based on morphology (Lienhard 1990; Li 1994, 2002). Key identification characteristics of *L. bostrychophila* are as follows: the body length was usually bigger than 1 mm. There are tubercles of medium to large size on the vertex and abdominal terga. The head was chocolate-colored with six or seven ommatidia. There was one pair of lateral setae in the posterior half of the prosternum, and no pronotal setae (*PNS*) in addition to humeral seta. Abdominal terga 3–7 each presented a pale posterior membranous band—this is known as an abdomen of the “annulate type.” Only the dorsal and ventral marginal seta of tergum 10 (*Md10* and *Mv10*) differentiated, whereas the marginal seta of tergum 8 (*M*8), the ventral marginal seta of terum 9 (*M*9), and discal seta (*D*) could not.

To reconstruct the mt genomes of the six strains of *L.bostrychophila*, *cox1*, *rrnS*, and *rrnL* gene fragments were chosen as “anchors” to obtain mt genome sequences. We first sequenced the *cox1*, *rrnS*, and *rrnL* gene fragments using universal primer pairs. Then, long-PCR primers were designed to amplify the chromosomes where the gene fragments were located. Total DNA was extracted from individual booklouse with a DNeasy Blood and Tissue Kit (Qiagen) following the manufacturer’s instructions. The mt *cox1*, *rrnS*, and *rrnL* gene fragments of the six strains of *L. bostrychophila* above were amplified with the primer pairs LCO1490–HCO2198 (Folmer *et al.* 1994), 12SF–12SR (Kambhampati and Smith 1995) and 16Sar–16Sbr (Simon *et al.* 1994) following the methods described by Yang *et al.* (2013, 2015). We then designed specific long-PCR primers based on the sequences of the three genes for each strain of *L. bostrychophila* (Supplemental Material, Table S1 and Table S2).

**Figure 1:**
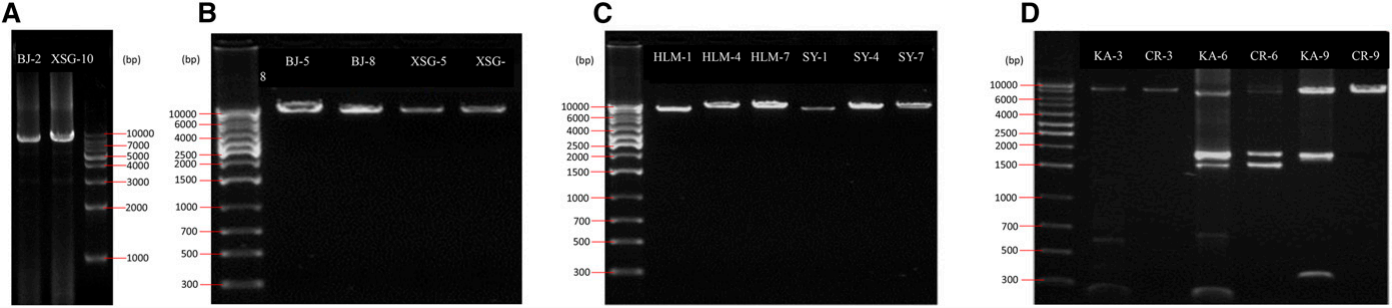
PCR amplification of mitochondrial DNA of *L. bostrychophila* collected from BJ and XSG (A and B); HLM and SY (C); and CR and KA (D) strains. The lanes are labelled with “Collection location name-primer code.” See Table S2 for primer codes. DNA marker: 1 kb DNA Ladder (Tiangen), 1 kb ladder DNA Marker (Axygen).

Each long-PCR amplification was performed in 25 μl containing 0.125 μl TaKaRa LA *Taq* (5 U/μl), 2.5 μl 10× LA *Taq* Buffer, 4 μl dNTP mixture, 2.5 μl MgCl_2_, 1 μl of each primer (10 μM), 1 μl genomic DNA, and 12.875 μl ddH_2_O. PCR cycling conditions were: 94° for 1 min, followed by 40 cycles of 98° for 10 sec, 68° for 9–15 min, and, finally, 72° for 6 min. The size of PCR amplicons was estimated by agarose gel electrophoresis (1%). Long-PCR amplicons were purified with a WizardGel and PCR Clean-Up System (Promega). The concentration and purity of purified amplicons were checked with Nanodrop. Long-PCR amplicons were sequenced at the BGI (Hong Kong) with Illumina Hisequation 2000 platform in both directions except for one amplicon (XSG-10, Figure 1) of XSG strain, which was sequenced using the Sanger method at the Sangon Biotech (Shanghai) with a primer walking strategy (Table S3). Illumina sequence-reads were mate-paired and 90 bp each. Sanger sequence-reads were 600 bp each.

### Sequence assembly and gene identification

Illumina sequence-reads were assembled with Geneious (Kearse *et al.* 2012) using the sequences of the three gene fragments (*rrnS*, *rrnL*, and *cox1*) as initial references (Table S1). The contigs were then extended using the assembly parameters: (1) minimum overlap 50 bp, and (2) minimum similarity 99%, until the full circular mt chromosome sequences were obtained. Chromosome II of XSG strain was assembled separately. The *rrnS*, *rrnL*, and *cox1* gene fragments of XSG strain were identical to those of BJ strain. So, chromosome II of XSG strain was assembled by using chromosome II of BJ strain as a reference. Protein-coding genes were identified by searching open reading frames (ORF) in Geneious, and then BLAST searches in GenBank. The two rRNA genes were also found by BLAST searches in GenBank. The tRNA genes were identified with tRNAscan-SE (Lowe and Eddy 1997) and ARWEN (Laslett and Canbäck 2008) based on secondary structures. All genes were confirmed by alignment with homologous genes from other booklice.

### Phylogenetic analysis

Five species of booklice were included in the phylogenetic analysis. The barklouse *Psococerastis albimaculata* was used as an outgroup (Li *et al.* 2013). Sequences reported in previous studies were retrieved from GenBank (Table S4). Sequences of the 12 protein-coding genes (*cox1*, *cox2*, *cox3*, *nad1*, *nad2*, *nad3*, *nad4*, *nad5*, *nad6*, *atp6*, *atp8*, and *cob*) were used in the analysis. Sequences were aligned using ClustalW with the default parameters implemented in MEGA 5.0 (Tamura *et al.* 2011). The ambiguous positions in the alignment of genes were filtered using GBlocks 0.91b (Castresana 2000) with default settings. jModelTest 2.1.7 (Posada 2008) was used to find a suitable model for nucleotide substitution, and GTR+I+G model was chosen for both datasets. Two datasets were used to build phylogenetic trees: (1) PCG123: 12 protein-coding genes (all three codon positions included) with 9021 nucleotides and (2) PCG12: 12 protein-coding genes (third codon positions excluded) with 6014 nucleotides.

Maximum likelihood (ML) analysis was run with RAxML-HPC2 8.1.11(Stamatakis 2006). Bootstrapping analysis with 1000 replicates was performed with the fast likelihood-based method implemented in RAxML. MrBayes 3.2.5 (Ronquist and Huelsenbeck 2003) was used for Bayesian inference (BI) analysis. Two independent runs with four simultaneous Markov chains were run for 10,000,000 generations, and trees were sampled every 1000 generations. Majority-rule consensus trees were estimated combining results from duplicated analysis, with the first 25% generations discarded.

Concordance analysis was conducted to test the concatenation of all mt genes for phylogenetic analyses using BUCKy (Ané *et al.* 2007; Larget *et al.* 2010), which took the files generated by MrBayes for all 14 mt genes and output a greedy consensus tree. A concordance factor (CF) was calculated for each clade of two datasets, and revealed the proportion of genes that supported the clade. Cryptic species is indicated if *L. bostrychophila* strains are in different clades.

### Data availability

The authors state that all data necessary for confirming the conclusions presented in the article are represented fully within the article.

## Results

### The mitochondrial genomes of six asexual strains of L. bostrychophila

**Figure 2:**
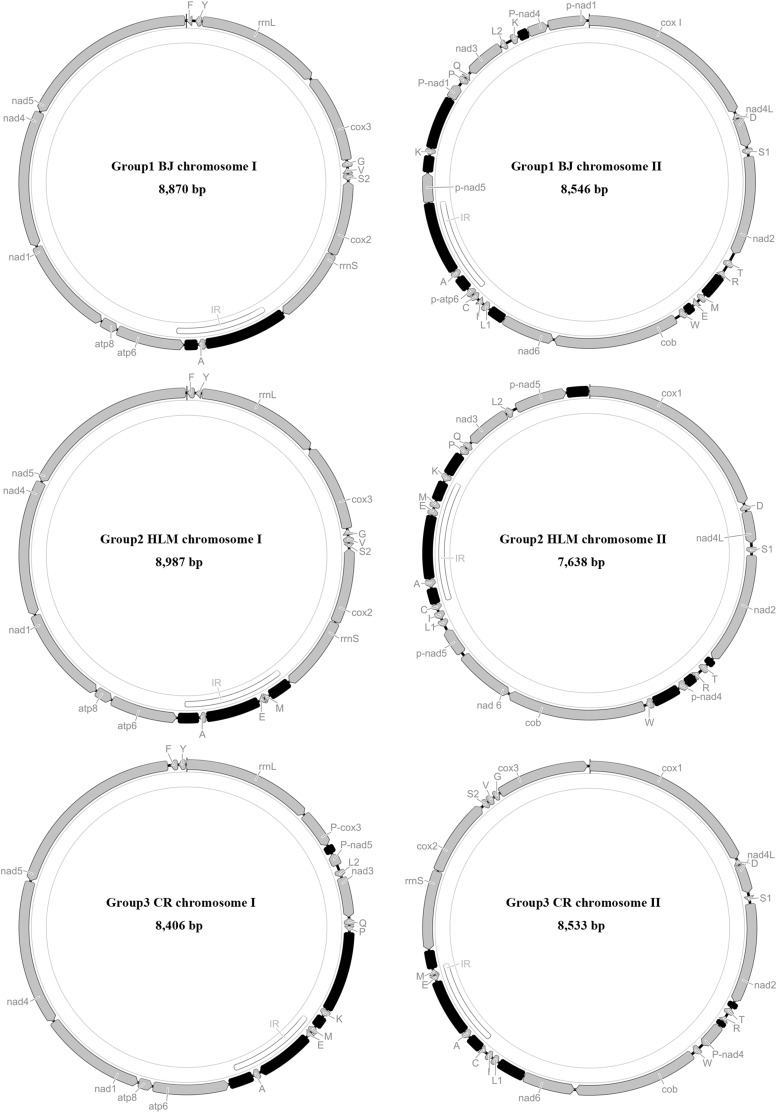
The mitochondrial genome organizations of three groups of *L. bostrychophila*. Circular maps were drawn with Geneious (Kearse *et al.* 2012). The transcriptional direction is indicated with arrows. Coding genes are shown in gray, noncoding regions in black, the identical region between the two chromosomes in white. *cox1–3*, cytochrome oxidase subunits 1–3; *cob*, cytochrome b; *nad1–6* and *nad4L*, NADH dehydrogenase subunits 1–6 and 4L; *rrnL* and *rrnS*, large and small rRNA subunits; *atp6* and *atp8*, ATP synthase subunits 6 and 8. tRNA genes are indicated with their one-letter corresponding amino acids.

We obtained three long-PCR amplicons for each of the six asexual strains of *L. bostrychophila* with the specific primers designed from *rrnS*, *rrnL*, and *cox1* sequences (Figure 1). Each long-PCR amplicon is 7–9 kb in size. The mt genomes of all six asexual strains of *L. bostrychophila* consist of two chromosomes (I and II). The gene content and gene arrangement in these chromosomes, however, differed among the six strains (Figure 2). The mt chromosomes assembled from the *rrnS* amplicon (*i.e.*, obtained with *rrnS* specific primers) and *rrnL* amplicon (obtained with *rrnL* specific primers) were identical in sequence for BJ, XSG, HLM, and SY strains, respectively, whereas the chromosomes assembled from the *rrnS* amplicon and *cox1* amplicon (obtained with *cox1*-specific primers) were identical for CR and KA strains, respectively. We identified 35 of the 37 genes commonly seen in animals, including 20 tRNA genes (Figure S1); we were unable to find two tRNA genes (*trnN* and *trnH*) in any of these six strains.

The mt genomes of the BJ strain and XSG strain (Group 1) have a 8870-bp circular chromosome (I) with 15 genes, and a 8546-bp circular chromosome (II) with 22 genes (Table S5 and Table S6); 20 tRNA genes were found in these two strains, and two tRNA genes (*trnA* and *trnS*) were found in both chromosomes. An identical 881-bp region (including noncoding regions, *trnA*, and partial *nad6*) was present in both chromosomes. These two strains share the same gene content and gene arrangement in both chromosomes, and have high sequence similarity (chromosome I: 99.9%; chromosome II: 100%).We named BJ strain and XSG strain after Group 1 based on their sequence consistency.

The mt genomes of HLM strain and SY strain (Group 2) have a 8987-bp circular chromosome (I) with 17 genes and a 7638-bp circular chromosome (II) with 21 genes (Table S7 and Table S8); 20 tRNA genes were found in these two strains, and three tRNA genes (*trnA*, *trnM*, and *trnE*) were found in both chromosomes. A 986-bp region (including noncoding regions, *trnA*, *trnM*, and *trnE*) was present in both chromosomes. These two strains share the same gene content and gene arrangement in both chromosomes, and have high sequence similarity (chromosome I: 99.9%; chromosome II: 99.9%). HLM strain and SY strain were called Group 2.

Group 3 included KA strain and CR strain. The mt genome of KA strain has a 7932-bp circular chromosome (I) with 16 genes and a 8530-bp circular chromosome (II) with 22 genes. The mt genome of CR strain has a 8406-bp circular chromosome (I) with 16 genes and a 8533-bp circular chromosome (II) with 22 genes (Table S9 and Table S10). These two strains have the same gene content and gene arrangement in both chromosomes except that chromosome I of CR strain has an additional 477-bp noncoding region. Excluding the 477-bp region, the two strains have 99.7% sequence similarity between their chromosomes I, and they have 99.7% sequence similarity between their chromosomes II. Twenty tRNA genes were found in these two strains; three tRNA genes (*trnA*, *trnL*, and *trnE*) were found in both chromosomes of each strain. A 945-bp region (including noncoding regions, *trnA*, *trnM*, and *trnE*) was present in both chromosomes of the KA strain, and a 808-bp region (including noncoding regions, *trnA*, *trnM*, and *trnE*) in both chromosomes of the CR strain. These two strains share the same gene content and gene arrangement in both chromosomes with the BB strain reported previously (Wei *et al.* 2012); KA strain and CR strain, together with the published BB strain, formed Group 3. The sequences of the mt genomes of the six asexual strains of *L. bostrychophila* generated in the current study were deposited in GenBank under accession numbers KY656890–KY656901.

### Common gene clusters in the asexual strains of L. bostrychophila

**Figure 3:**
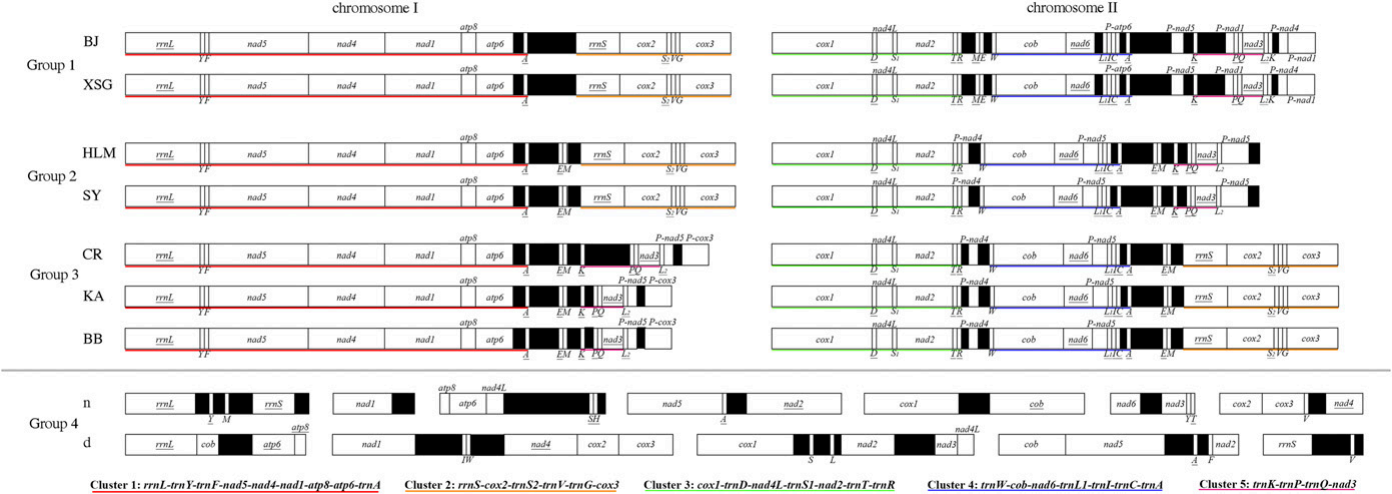
Arrangement of mt genes in four Groups of *L. bostrychophila*. The circular mt genomes have been linearized for ease of comparison. Gene names are the standard abbreviations used in this study. The gene cluster 1 is in red, cluster 2 in orange, cluster 3 in green, cluster 4 in blue, and cluster 5 in purple.

Five gene clusters were found in all of the asexual strains of *L. bostrychophila*: (1) *rrnL-trnY-trnF-nad5-nad4-nad1-atp8-atp6-trnA*, (2) *rrnS-cox2-trnS2-trnV-trnG-cox3*, (3) *cox1-trnD-nad4L-trnS1-nad2-trnT-trnR*, (4) *trnW-cob-nad6-trnL1-trnI-trnC-trnA*, and (5) *trnK-trnP-trnQ-nad3* (Figure 3). The distribution of these gene clusters between the two mt chromosomes, however, differs among the asexual strains of *L. bostrychophila*. Group 1 and Group 2 have the same distribution pattern of the five gene clusters: clusters 1 and 2 are on chromosome I; clusters 3, 4, and 5 are on chromosome II. In Group 3, however, clusters 1 and 5 are on chromosome I; clusters 3, 4, and 2 are on chromosome II. These clusters are not present in the two sexual strains of *L. bostrychophila*.

### Phylogeny of booklice inferred from mt genome sequences

**Figure 4:**
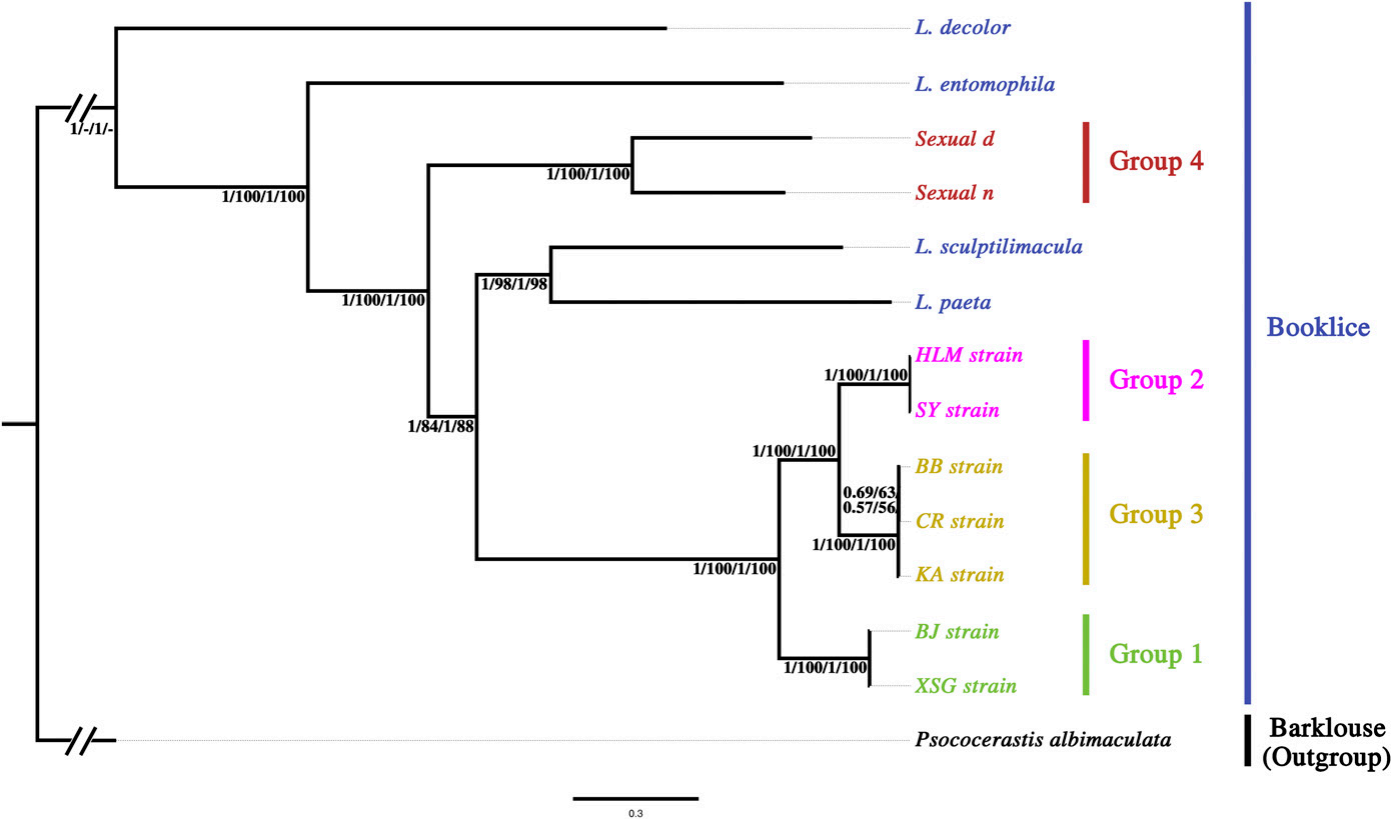
BI and ML phylogenetic trees inferred from mt genomes of booklice. Numbers above or below the branches show support for tree nodes from nucleotide sequences of the two datasets: Bayesian posterior probability of PCG123, ML bootstrap support values of PCG123, Bayesian posterior probability of PCG12, ML bootstrap support values of PCG12. Group 1 is in green, Group 2 in pink, Group 3 in brown, Group 4 in red, other species of booklice in blue, the outgroup in black.

Phylogenetic trees were constructed using the mt genome sequences of nine strains of *L. bostrychophila* and four other *Liposcelis* species with BI and ML methods (Figure 4). The barklouse, *Psococerastis albimaculata*, was used as the outgroup. The seven asexual strains of *L. bostrychophila* were clustered together and could be divided into three groups: BJ and XSG strains in Group 1; HLM and SY strains in Group 2; and CR, KA and BB strains in Group 3. The two sexual strains were clustered in Group 4. There is strong support for each group of *L. bostrychophila* (posterior probability 1, bootstrap value 100%). Unexpectedly, the asexual strains of *L. bostrychophila* were not clustered with the sexual strains, but were grouped with two different species, *L. paeta* and *L. sculptilimacula*. The sexual strains of *L. bostrychophila* were sister to the clade that contains the asexual *L. bostrychophila*, *L. paeta*, and *L. sculptilimacula*. The topology of the primary concordance tree from BUCKy was the same as ML and BI phylogenetic trees, except that the concordance tree supported KA strain and CR strain as a clade while ML and BI trees supported BB strain and CR strain as a clade (Figure S2). This difference, however, only changed the phylogenetic relationships among KA, CR, and BB strains, which all belonged to Group 3; it did not change relationships among different groups of *L. bostrychophila*.

## Discussion

### Rapid evolution of mt genomes in L. bostrychophila

There is substantial variation in mt genome organization within *L. bostrychophila* (as defined currently as a single species). First, each of the seven asexual strains investigated in the present study and by Wei *et al.* (2012) has two mt chromosomes (Table 1); the two sexual strains, however, have five and seven mt chromosomes each (Perlman *et al.* 2015). Second, five gene clusters, which contain 33 genes in total, are present in all of the asexual strains but are not present in the sexual strains. Third, the distribution of these gene clusters in the two mt chromosomes of each asexual strain differs among the three groups (Figure 3). For Group 1 and Group 2, the first two gene clusters are on chromosome I, whereas the other three gene clusters are on chromosome II. For Group 3, the gene clusters 1 and 5 are on chromosome I whereas the gene clusters 2, 3, and 4 are on chromosome II. As Group 2 and Group 3 are phylogenetically more closely related to each other than either of them is to Group 1 (Figure 4), we infer that the common distribution pattern between Group 1 and Group 2 is ancestral to the asexual strains of *L. bostrychophila*, whereas the distribution pattern in Group 3 is derived. To account for the variation among the asexual groups, a swap between clusters 2 and 5 is required to occur in Group 3 after the divergence of Group 2 and Group 3, likely via a recombination event between chromosomes I and II. Forth, the three asexual groups are different from each other in the copy number and position of *trnE-trnM*. In Group 1, this two-gene cluster is present once on chromosome II between gene clusters 3 and 4. In Group 2 and Group 3, however, *trnE-trnM* is duplicated. In Group 2, one copy of *trnE-trnM* is between gene clusters 1 and 2 on chromosome I, and another copy is between gene clusters 4 and 5 on chromosome II. In Group 3, one copy of *trnE-trnM* is between gene clusters 1 and 5 on chromosome I, and another copy is between gene clusters 2 and 4 on chromosome II (Figure 3). There is also substantial sequence divergence among the four groups of *L. bostrychophila*. For protein-coding and rRNA genes, the lowest pair-wise sequence similarity is 48.7% (*nad6*, between Group 1 and Group 4d) and the highest similarity is 87.4% (*rrnS*, between Group 2 and Group 3) (indicated in bold in Table 2). Between the two sexual strains, the lowest pair-wise sequence similarity is 48.9% (*nad6*) and the highest is 79.4% (*rrnS*).

**Table 1:** Comparison of mitochondrial genomes of different groups of asexual *Liposcelis bostrychophila*

Chromosome		Group I		Group II		Group III		
		BJ	XSG	HLM	SY	KA	CR	BB
Length	Chromosome I	8870	8870	8987	8987	7932	8406	7933
	Chromosome II	8546	8546	7638	7639	8530	8533	8530
A+T%	Chromosome I	68.8	68.9	69.7	69.7	69.4	69.1	69.3
	Chromosome II	69.6	69.6	69.5	69.5	67.8	67.9	67.8
A%	Chromosome I	29.5	29.5	30.2	30.2	39.1	38.6	39.2
	Chromosome II	31.6	31.6	31.1	31.1	30	30.1	30.1
T%	Chromosome I	39.3	39.3	39.5	39.5	30.3	30.5	30.4
	Chromosome II	38.1	38.1	38.4	38.4	37.8	37.8	37.7
G%	Chromosome I	13.1	13.1	12.6	12.6	18.1	18.3	18.1
	Chromosome II	13.4	13.4	13.3	13.3	13.8	13.8	13.9
C%	Chromosome I	18	18	17.7	17.7	12.4	12.6	12.4
	Chromosome II	17	17	17.2	17.2	18.3	18.3	18.3
AT-skew	Chromosome I	−0.142	−0.142	−0.133	−0.133	0.126	0.117	0.126
	Chromosome II	−0.093	−0.093	−0.105	−0.105	−0.115	−0.113	−0.113
GC-skew	Chromosome I	−0.158	−0.158	−0.167	−0.167	0.187	0.182	0.186
	Chromosome II	−0.12	−0.12	−0.128	−0.126	−0.14	−0.141	−0.14
Total gene number	Chromosome I	15	15	17	17	16	16	16
	Chromosome II	22	22	21	21	22	22	22
Protein coding gene	Chromosome I	7	7	7	7	6	6	6
	Chromosome II	6	6	6	6	7	7	7
tRNA gene	Chromosome I	6	6	8	8	9	9	9
	Chromosome II	16	16	15	15	14	14	14
rRNA gene	Chromosome I	2	2	2	2	1	1	1
	Chromosome II	0	0	0	0	1	1	1

**Table 2:** Sequence similarities among different groups of *Liposcelis bostrychophila*

	*rrnS*	*rrnL*	*cox1*	*cox2*	*cox3*	*cob*	*atp6*	*atp8*	*nad1*	*nad2*	*nad3*	*nad4*	*nad4L*	*nad5*	*nad6*
Group 1-Group 2	0.805	0.78	0.82	0.76	0.77	0.79	0.764	0.705	0.759	0.697	0.716	0.737	0.725	0.726	0.653
Group 1-Group 3	0.815	0.76	0.81	0.81	0.779	0.776	0.763	0.705	0.76	0.696	0.707	0.748	0.733	0.739	0.684
Group 1-Group 4d	0.596	0.607	0.709	0.649	0.612	0.671	0.577	0.528	0.62	0.512	0.608	0.581	0.604	0.556	**0.487**
Group 1-Group 4n	0.611	0.615	0.705	0.629	0.671	0.682	0.594	0.522	0.623	0.514	0.605	0.58	0.6	0.548	0.57
Group 2-Group 3	**0.874**	0.854	0.844	0.779	0.826	0.844	0.803	0.821	0.81	0.799	0.809	0.83	0.797	0.808	0.71
Group 2-Group 4d	0.622	0.599	0.705	0.635	0.615	0.667	0.582	0.56	0.636	0.522	0.584	0.581	0.592	0.547	0.505
Group 2-Group 4n	0.62	0.599	0.703	0.626	0.642	0.677	0.605	0.522	0.653	0.52	0.558	0.585	0.613	0.552	0.532
Group 3-Group 4d	0.602	0.597	0.713	0.638	0.605	0.663	0.589	0.585	0.625	0.512	0.598	0.581	0.542	0.55	0.505
Group 3-Group 4n	0.603	0.598	0.712	0.612	0.662	0.682	0.59	0.535	0.635	0.535	0.558	0.582	0.596	0.549	0.52
Group4d-Group 4n	0.794	0.794	0.766	0.74	0.713	0.768	0.756	0.654	0.763	0.74	0.774	0.724	0.759	0.713	0.489

Comparison of gene sequence similarities among four groups of rRNA genes and protein coding genes. Gene names are the standard abbreviations used in this study. The sequence similarity inside the asexual groups is high. So, we chose one species representing for its Group: BJ for Group 1, HLM for Group 2 and CR for Group 3. But the sequence similarity between two sexual *L. bostrychophila* of Group 4 is low. Thus, we put them both in the comparisons, Group 4d for sexual distort and Group 4n for sexual normal. The “Group n-Group m” means the sequence of Group n comparing with Group m. For protein-coding and rRNA genes, the lowest pair-wise sequence similarity is 48.7% (nad6, between Group 1 and Group 4d) and the highest similarity is 87.4% (rrnS, between Group 2 and Group 3) (indicated in bold).

The variation in mt genome organization and sequence within *L. bostrychophila* is unprecedented for an animal. Even when we consider *L. bostrychophila* as a cryptic species, *i.e.*, the sexual strains from Arizona and the asexual strains are two different species, the variation, both between the two sexual strains and among the seven asexual strains, is far beyond what we would expect within an animal species. It is puzzling why mt genomes evolved so fast in *L. bostrychophila*. As defined now, *L. bostrychophila* is the only species known so far in the genus *Liposcelis* that has both asexual and sexual reproductive modes. It would be tempting to think that rapid evolution of mt genomes might be linked to the unusual reproduction modes in *L. bostrychophila*. However, this may not be the case as four other *Liposcelis* species that reproduce only sexually also differ in mt genome organization. *L. entomophila* and *L. paeta* have bipartite mt genomes, whereas *L. decolor* and *L. sculptilimacula* have the typical single-chromosome mt genomes (Shi *et al.* 2016). Parasitic lice, which only reproduce sexually, and are most closely related to *Liposcelis* booklice, also have mt genomes that evolved much faster than most animals (Shao *et al.* 2015).

### L. bostrychophila as a cryptic species

*L. bostrychophila* is an important pest, found mainly in houses, grain depots, libraries, and other indoor places. For a very long time, it was thought that *L. bostrychophila* had females only and reproduced via thelytokous parthenogenesis (Goss 1954; Turner 1994). The first sexual strain of *L. bostrychophila* with both males and females was reported from Hawaii in 2008 (Mockford and Krushelnycky 2008). Two more sexual strains were reported from the Chiricahua Mountains of southeastern Arizona; these two strains coexisted in the same colony. One strain, called the normal strain, produced both male and female offspring; the other strain, called the distort strain, produced only female offspring. Naturally, the two sexual strains lived together, meaning that the normal strain shared its males with the distort strain (Perlman *et al.* 2015).

Our phylogenetic analyses of the mt genome sequences of booklice do not support the nine strains of *L. bostrychophila* as a single species. The seven asexual strains of *L. bostrychophila* form a monophyletic group, which corresponds to the *L. bostrychophila* species defined initially by Badonnel (1931). The two sexual strains of *L. bostrychophila* (from Arizona) are separated from the asexual strains of *L. bostrychophila* by two other species of booklice, *L. paeta* and *L. sculptilimacula* on our phylogenetic trees. Our results provided evidence for the first time that *L. bostrychophila*, as defined currently, is a cryptic species, *i.e.*, two or more species under one specie name that are indistinguishable morphologically (Beheregaray and Caccone 2007). Our results support the view that the sexual strains of *L. bostrychophila* (from Arizona) and the asexual strains of *L. bostrychophila* are two different species.

Mt genome analysis provided a phylogenetic framework for validating the species status of *L. bostrychophila*. However, the mt genome alone may not be sufficient for us to conclude that *L. bostrychophila* is indeed a cryptic species due to the different inheritance mode and evolutionary history of mt genomes from nuclear genomes (Rubinoff *et al.* 2006; Sehrish *et al.* 2015; Sloan *et al.* 2017). The novel results we obtained in this study need to be validated using nuclear gene analyses. In addition, the sexual strain of *L. bostrychophila* found in Hawaii was not available to us, thus was not included in the current study. It would be interesting to investigate how the Hawaii sexual strain of *L. bostrychophila* is related to the Arizona sexual strains and the asexual strains of *L. bostrychophila*.

## Supplementary Material

Supplemental material is available online at www.g3journal.org/lookup/suppl/doi:10.1534/g3.117.300410/-/DC1.
