# Supplementary material for: The Highly Divergent Mitochondrial Genomes Indicate That the Booklouse, Liposcelis bostrychophila (Psocoptera: Liposcelididae) Is a Cryptic Species
Source: G3 (Bethesda). 2018 Jan 19;8(3):1039–47. doi: 10.1534/g3.117.300410 (PMC5844292; doi:10.1534/g3.117.300410)
Supplement: Supplementary file 1 [file 1039FigureS1.pdf]

Figure S1. Inferred secondary structure of 20 tRNAs of *Liposcelis bostrychophila* of Group1, Group2 and Group3.

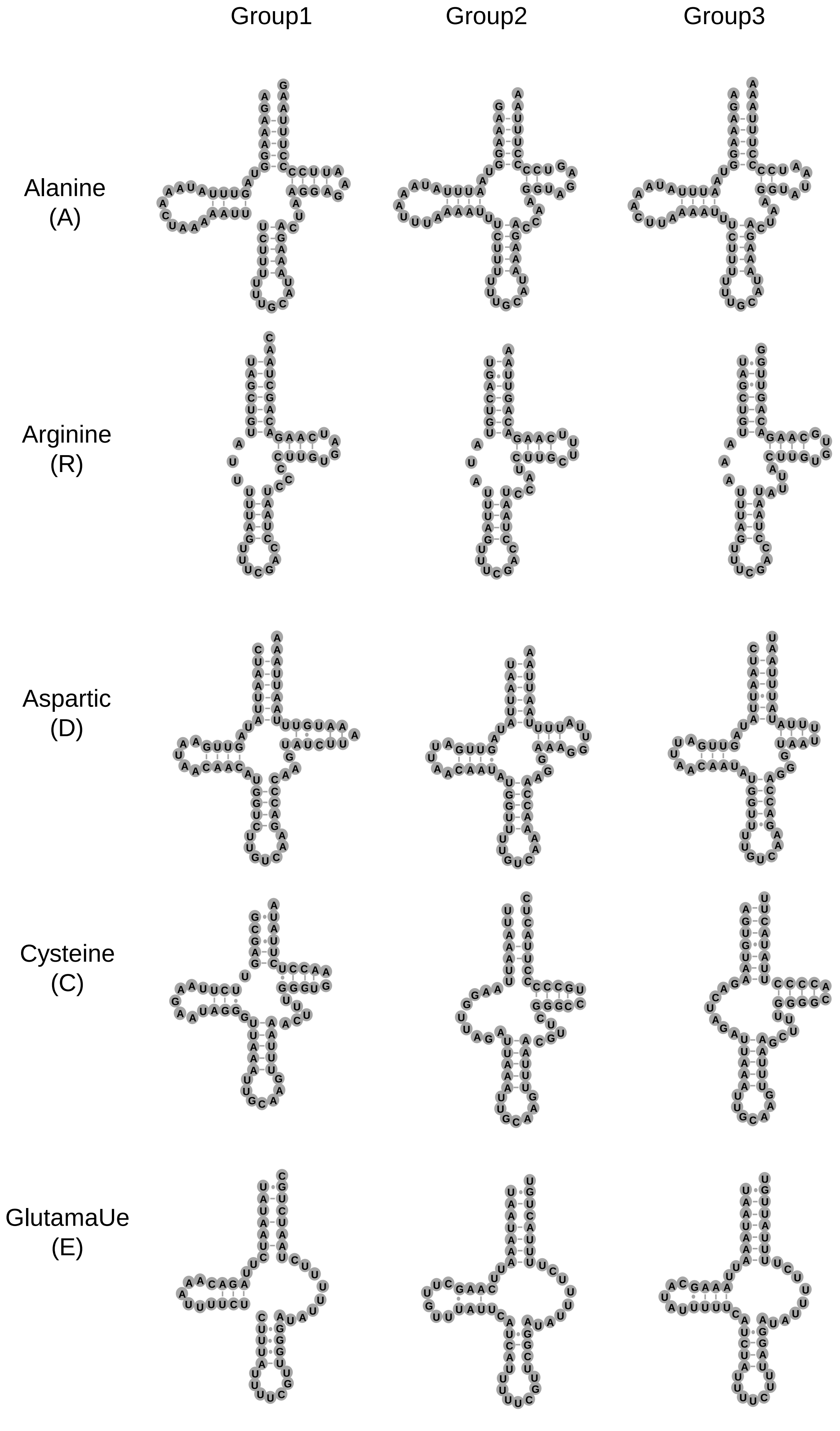

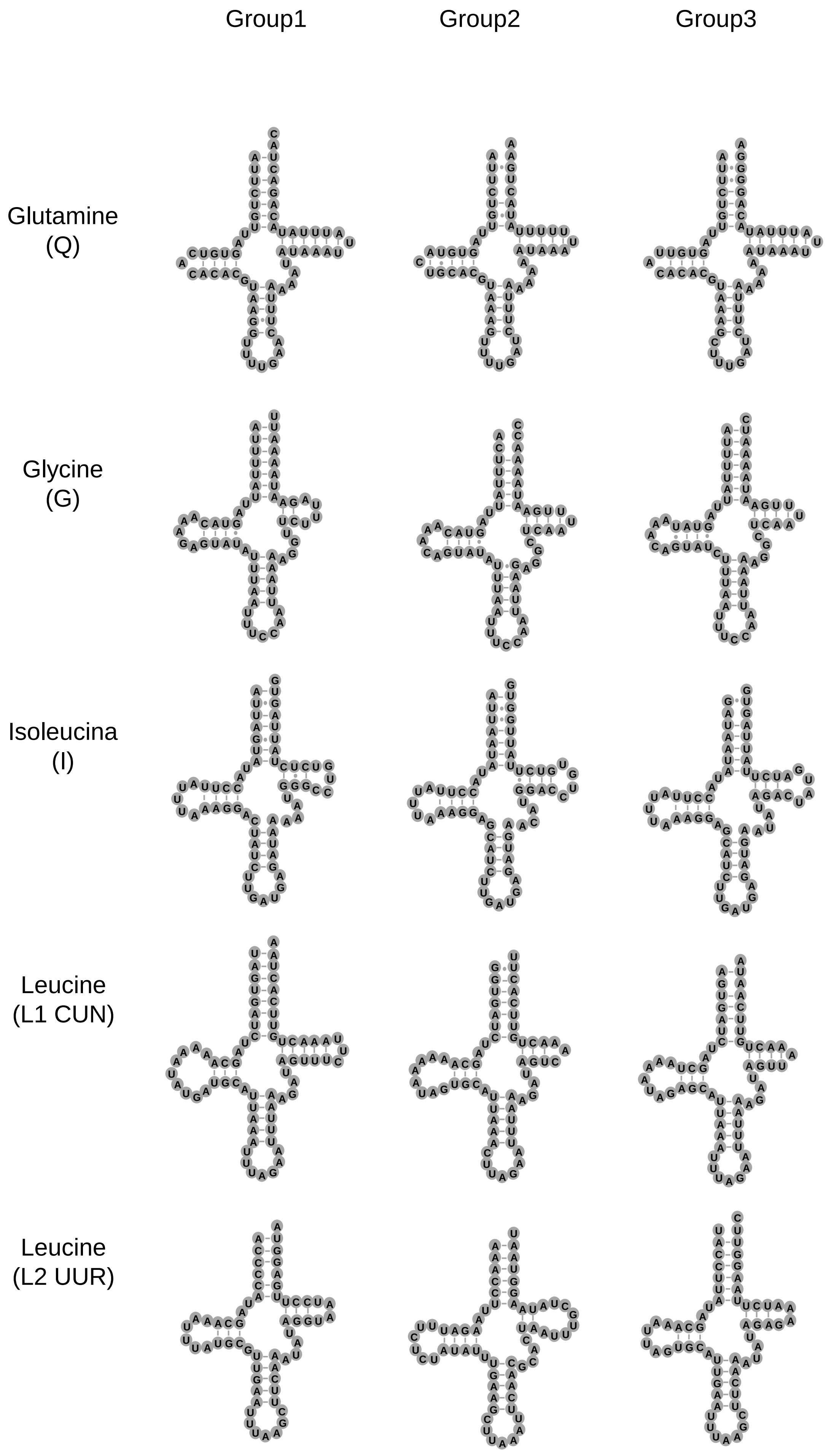
Group1
Group2
Group3
Glutamine
(Q)
Glycine
(G)
Isoleucina
(I)
Leucine
(L1 CUN)
Leucine
(L2 UUR)

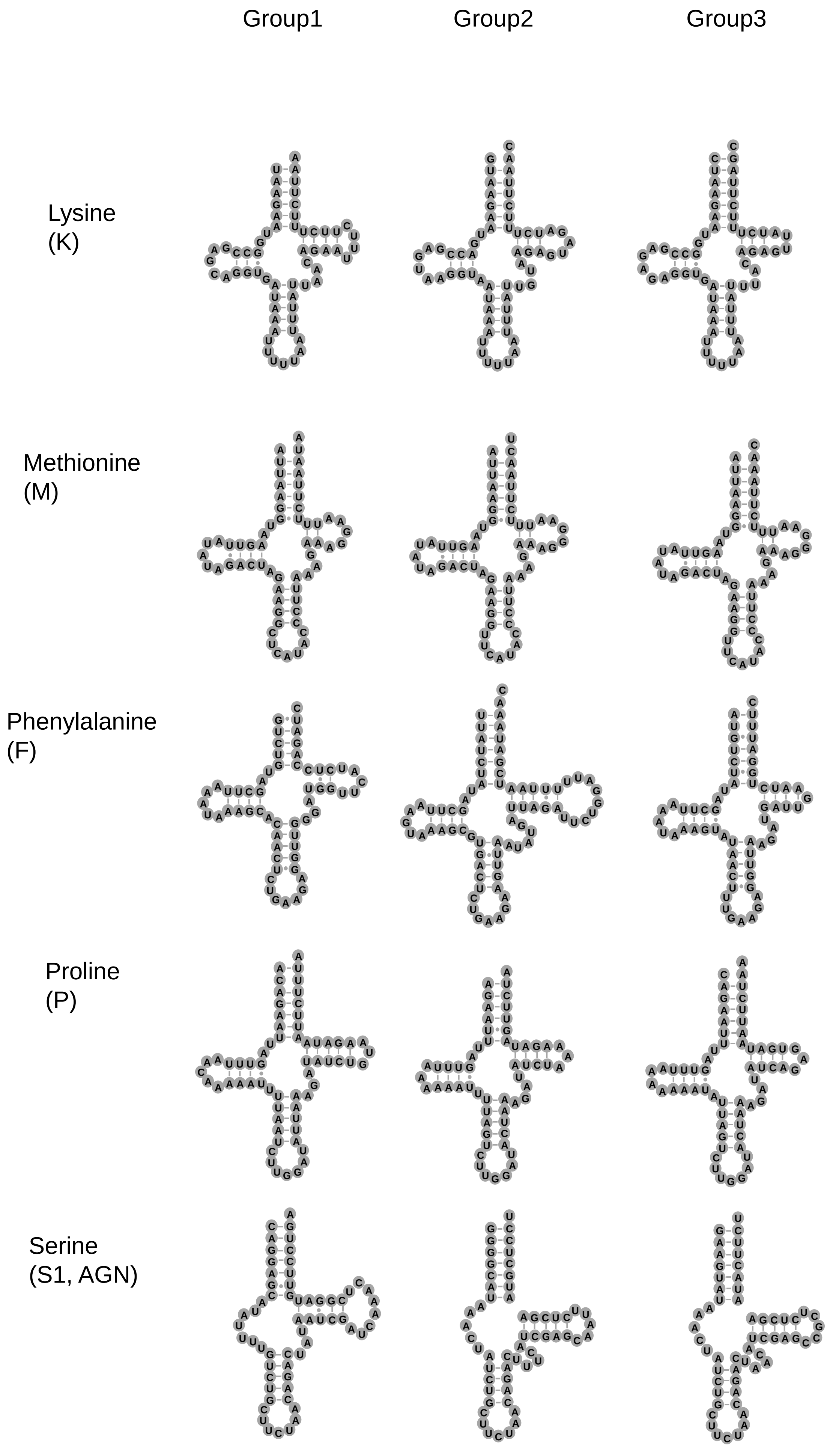
Group1
Group2
Group3
Lysine
(K)
Methionine
(M)
Phenylalanine
(F)
Proline
(P)
Serine
(S1, AGN)

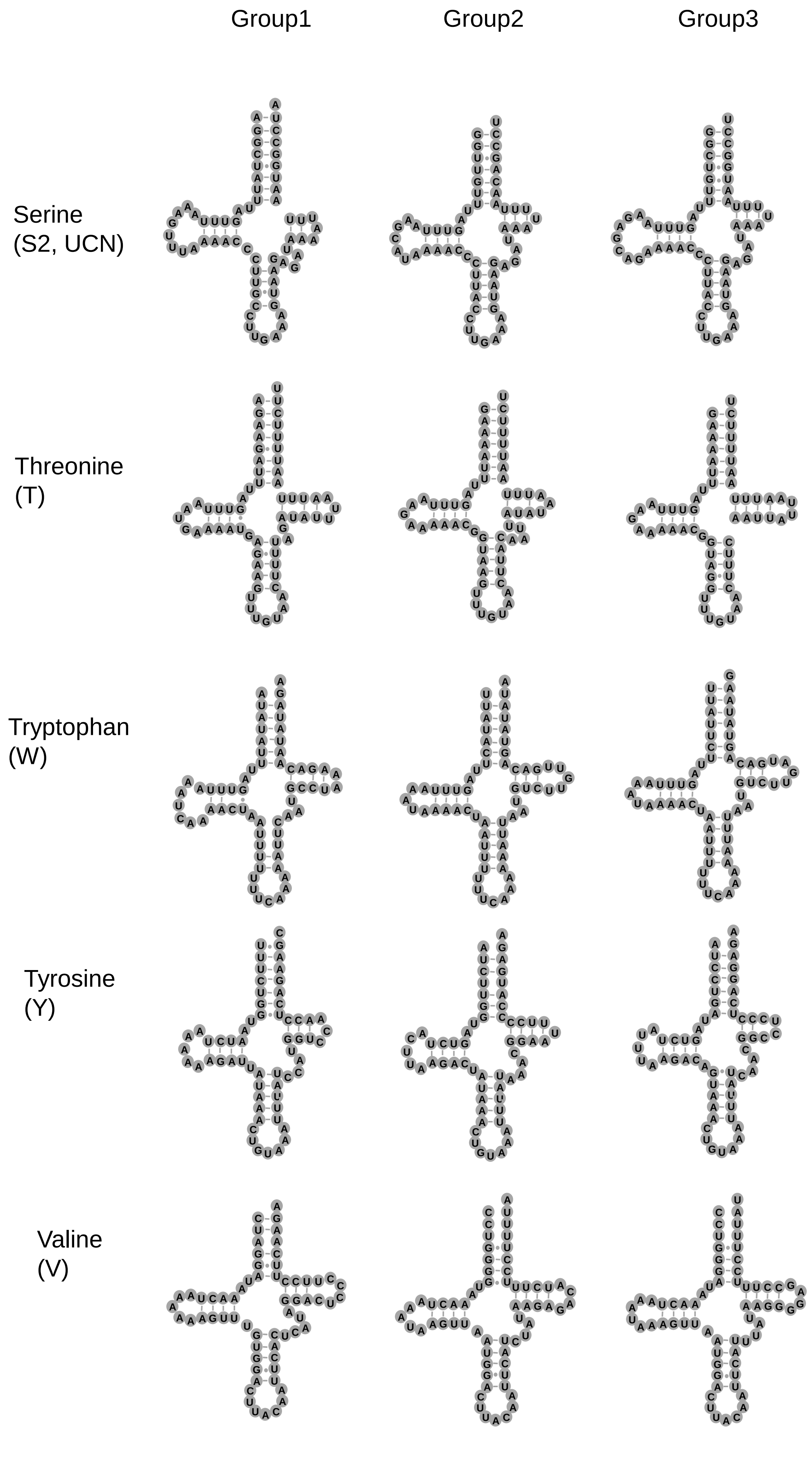
Group1
Group2
Group3
Serine (S2, UCN)
Threonine (T)
Tryptophan (W)
Tyrosine (Y)
Valine (V)

	Group1	Group2	Group3
Lysine (K2)	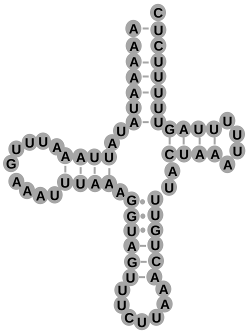		
