# Supplementary figures and images for: The Highly Divergent Mitochondrial Genomes Indicate That the Booklouse, Liposcelis bostrychophila (Psocoptera: Liposcelididae) Is a Cryptic Species
Source: G3 (Bethesda). 2018 Jan 19;8(3):1039–47. doi: 10.1534/g3.117.300410 (PMC5844292; doi:10.1534/g3.117.300410)

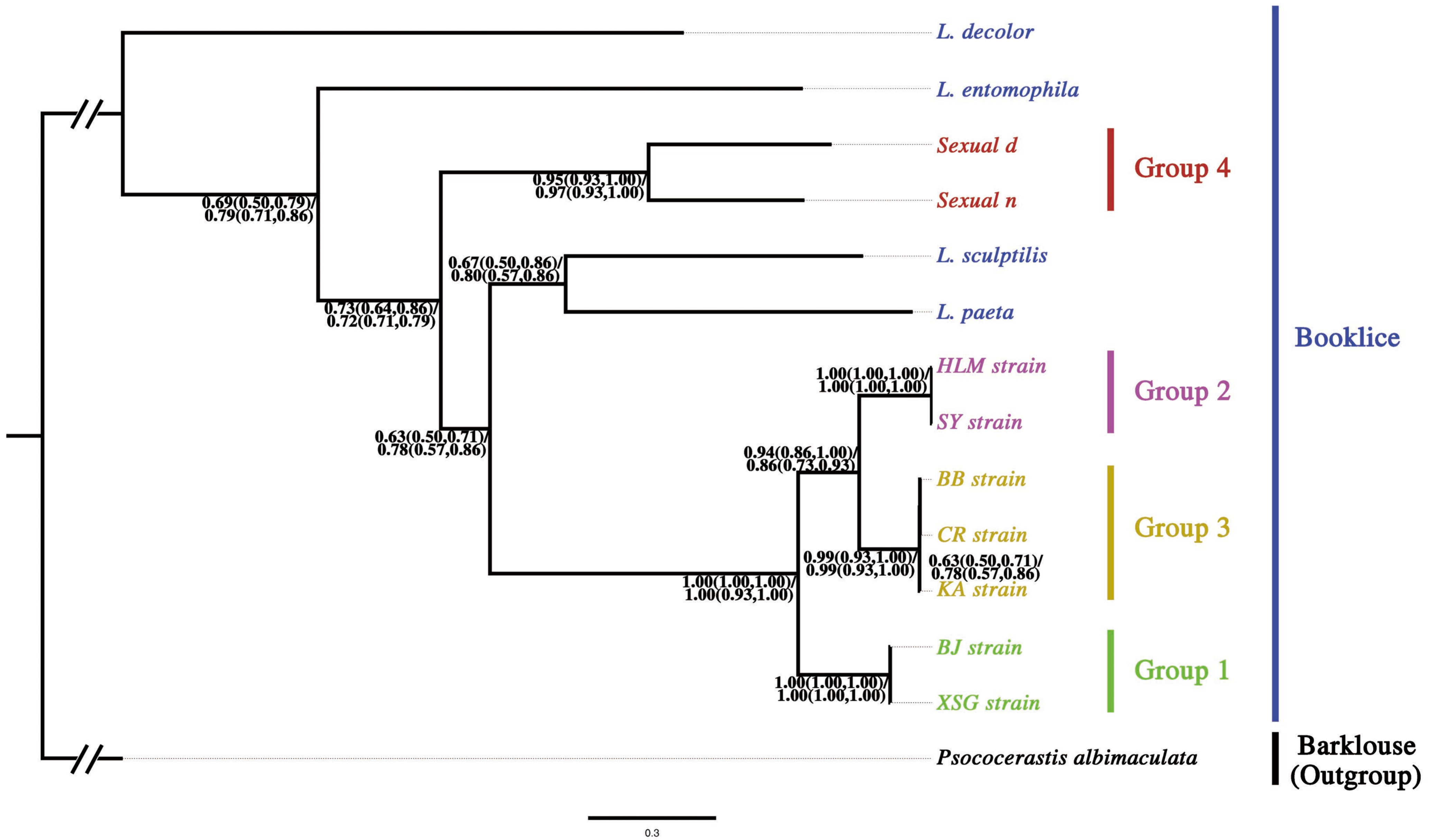

Supplement: Supplementary file 2 [file 1039FigureS2.pdf]
