# Supplementary material for: The Highly Divergent Mitochondrial Genomes Indicate That the Booklouse, Liposcelis bostrychophila (Psocoptera: Liposcelididae) Is a Cryptic Species
Source: G3 (Bethesda). 2018 Jan 19;8(3):1039–47. doi: 10.1534/g3.117.300410 (PMC5844292; doi:10.1534/g3.117.300410)
Supplement: Supplementary file 3 [file 1039TableS1.docx]

**Table S1.** *12S*, *16S*, *cox1* gene fragments of 6 strains of *Liposcelis bostrychophila*

| Strain | GenBank Accession Numbers | | |
| --- | --- | --- | --- |
|  | *12S* | *16S* | *cox1* |
| BJ | KY320016 | KY320022 | KY320028 |
| XSG | KY320017 | KY320023 | KY320029 |
| HLM | KY320018 | KY320024 | KY320030 |
| SY | KY320019 | KY320025 | KY320031 |
| KA | KY320020 | KY320026 | KY320032 |
| CR | KY320021 | KY320027 | KY320033 |
