# Supplementary material for: The Highly Divergent Mitochondrial Genomes Indicate That the Booklouse, Liposcelis bostrychophila (Psocoptera: Liposcelididae) Is a Cryptic Species
Source: G3 (Bethesda). 2018 Jan 19;8(3):1039–47. doi: 10.1534/g3.117.300410 (PMC5844292; doi:10.1534/g3.117.300410)
Supplement: Supplementary file 4 [file 1039TableS2.docx]

**Table S2.** Long-PCR primers used in this study

| Code | Gene | Name | Target strain | Primer sequence(5’-3’) | Length (bp) | GC% | Tm℃ |
| --- | --- | --- | --- | --- | --- | --- | --- |
| 1 | *cox1* | LbGXC1F | HLM,SY | CTGGCTGGAATTAGTTCTATCCTTGGGGCC | 30 | 53.3 | 68 |
|  |  | LbGXC1R | HLM,SY | AAACTGTCCATCCAGTTCCTGCACCAGGTC | 30 | 53.3 | 70.2 |
| 2 |  | LbCZC1F | BJ | TCAATCAATCTGTGAGTTGAGCCTCGTCGG | 30 | 53.3 | 66.4 |
|  |  | LbCZC1R | BJ | CTGAGTGAGCTTCAACGGCAGATAAGGGAG | 30 | 53.3 | 65.9 |
| 3 |  | LbCROC1F | KA,CR | GCTGGAATTAGTTCCATCCTGGGAGCCATC | 30 | 53.3 | 67.3 |
|  |  | LbCROC1R | KA,CR | GTTCCTGCGCCTGGTCCAACGATTATTCTG | 30 | 53.3 | 68 |
| 4 | *16S* | LbGXLF | HLM,SY | CCTGGTAGTTCAGGCCTGATTTAGTCTG | 28 | 50 | 63.7 |
|  |  | LbGXLR | HLM,SY | AAGATCCTAGGGTCTTCTCGTCCCTTTG | 28 | 50 | 63.4 |
| 5 |  | LbCZLF | BJ,XSG | GGGACGAGAAGACCCTAGGATCTTTACAAC | 30 | 50 | 64.6 |
|  |  | LbCZLR | BJ,XSG | AGCGGCTCTTTAATACTCAGTGAGCAGGGC | 30 | 53.3 | 67.2 |
| 6 |  | LbCROLF | KA,CR | GGGGACGAGAAGACCCTAGGATCTTTACAG | 30 | 53.3 | 65.6 |
|  |  | LbCROLR | KA,CR | CAGGGCCGACCCAAGAAATTAAAGAGGGAC | 30 | 53.3 | 66.5 |
| 7 | *12S* | LbGXSF | HLM,SY | CTCTAATTTGTAAGAGAGCCGCCACAGACC | 30 | 50 | 66.3 |
|  |  | LbGXSR | HLM,SY | AGAGAGTGTACATACCGCCCGTCACTTCCC | 30 | 56.7 | 68.1 |
| 8 |  | LbZZSF | BJ,XSG | GTCGGCGATATGGAGGATGAGAGAACTAAG | 30 | 50 | 64.4 |
|  |  | LbZZSR | BJ,XSG | CGTCACTTCCCCCCAAAGGAATAAGTCG | 28 | 53.6 | 65.1 |
| 9 |  | LbCROSF | KA,CR | GGTGGGTTGTCCATTAGGTTCATGGATCTC | 30 | 50 | 65.8 |
|  |  | LbCROSR | KA,CR | AAGAGAGTGTACATACCGCCCGTCACTTCC | 30 | 53.3 | 66.6 |
| 10 | *cob* | LbcobF | XSG | GGGGTTCAATGTTGGTGACCCCACTTTAAC | 30 | 50 | 66.3 |
|  |  | LbcobR | XSG | TACGGACGCCCCGGTAGAATGAATAAATCG | 30 | 50 | 65.5 |
