# Supplementary material for: The Highly Divergent Mitochondrial Genomes Indicate That the Booklouse, Liposcelis bostrychophila (Psocoptera: Liposcelididae) Is a Cryptic Species
Source: G3 (Bethesda). 2018 Jan 19;8(3):1039–47. doi: 10.1534/g3.117.300410 (PMC5844292; doi:10.1534/g3.117.300410)
Supplement: Supplementary file 5 [file 1039TableS3.docx]

**Table S3.** 11 pairs of primers for sequencing chromosome II of Xingshagang strain

| ID | | Primer sequence(5’-3’) |
| --- | --- | --- |
| 1 | F | TTTAGGATAATCATCGGGCC |
|  | R | GCCCTCAATTCACTCAATTC |
| 2 | F | GGTCTAAATTTCTGGTTGCC |
|  | R | TGATGTGGCCAAATTGAAAG |
| 3 | F | TCAGGTCAAGATAAAGACAG |
|  | R | ATGAGAGTCTCAAATGAACC |
| 4 | F | TATGCTGACCTTTACCTGAG |
|  | R | GAGAACATAGCCCAAGAAAG |
| 5 | F | GCTTGGCCTATGTTTATCAG |
|  | R | CTATAACGTCAAGGAGAGTG |
| 6 | F | AAAATTTCTGACGTCTTGAG |
|  | R | ATTTAGTGGCTTAGCTCCAC |
| 7 | F | TTCAACATTCTTCTTTCTTC |
|  | R | TATAGTCTTTAACATCAAGC |
| 8 | F | AGATATAAAGGCCCTAGCTC |
|  | R | AACTTTTCTTCCCGTAACTC |
| 9 | F | GTGCGACAAACATAGCAGAC |
|  | R | GAGCTTCAACGGCAGATAAG |
| 10 | F | ATGTTTGGAGTTTGGTCAGG |
|  | R | ATCCTCTCTTGAGAGATGAC |
| 11 | F | TACCTGACTCCAAGAAATAG |
|  | R | TTTACCAATATGGGCGTAAC |
