# Supplementary material for: The Highly Divergent Mitochondrial Genomes Indicate That the Booklouse, Liposcelis bostrychophila (Psocoptera: Liposcelididae) Is a Cryptic Species
Source: G3 (Bethesda). 2018 Jan 19;8(3):1039–47. doi: 10.1534/g3.117.300410 (PMC5844292; doi:10.1534/g3.117.300410)
Supplement: Supplementary file 6 [file 1039TableS4.docx]

**Table S4.** GenBank accession numbers of species and strains whose mitochondrial genome sequences were included in phylogenetic analyses.

| Species (Strains) | GenBank accession number |
| --- | --- |
| *Liposcelis bostrychophila* BJ strain | KY656890, KY656891 (This study) |
| *Liposcelis bostrychophila* XSG strain | KY656892, KY656893 (This study) |
| *Liposcelis bostrychophila* HLM strain | KY656894, KY656895 (This study) |
| *Liposcelis bostrychophila* SY strain | KY656896, KY656897 (This study) |
| *Liposcelis bostrychophila* KA strain | KY656898, KY656899 (This study) |
| *Liposcelis bostrychophila* CR strain | KY656900, KY656901 (This study) |
| *Liposcelis bostrychophila* BB strain | JN645275, JN645276 |
| *Liposcelis bostrychophila* sexual distort | KP657697- KP657699, KP671844, KP671845 |
| *Liposcelis bostrychophila* sexual normal | KP641133, KP657691- KP657696 |
| *Liposcelis paeta* | KF649225, KF649226 |
| *Liposcelis decolor* | JX870621.1 |
| *Liposcelis entomophila* | KF649223, KF649224 |
| *Liposcelis sculptilis* | KX171073.1 |
| *Psococerastis albimaculata* | JQ910989.1 |
