# Supplementary material for: The Highly Divergent Mitochondrial Genomes Indicate That the Booklouse, Liposcelis bostrychophila (Psocoptera: Liposcelididae) Is a Cryptic Species
Source: G3 (Bethesda). 2018 Jan 19;8(3):1039–47. doi: 10.1534/g3.117.300410 (PMC5844292; doi:10.1534/g3.117.300410)
Supplement: Supplementary file 7 [file 1039TableS5.docx]

**Table S5.** Chromosome I of *Liposcelis bostrychophila* collected from Beijing (Group 1).

| **Gene^a^** | **Region** | **Size (bp)** | **GC%** | **Start codon** | **Stop codon** | **Anticodon** |
| --- | --- | --- | --- | --- | --- | --- |
| ***nad5*** | 1-1572 | 1572 | 31.6% | ATG | TAA |  |
| ***nad4*** | 1572-2780 | 1209 | 29.6% | ATT | TAA |  |
| ***nad1*** | 2780-3653 | 874 | 33.8% | ATT | T |  |
| ***atp8*** | 3654-3809 | 156 | 39.1% | GTG | TAG |  |
| ***atp6*** | 3775-4410 | 636 | 31.1% | ATA | TAG |  |
| ***IR*** | 4335-5215 | 881 | 29.9% |  |  |  |
| ***NCRI1*** | 4411-4531 | 121 | 28.9% |  |  |  |
| ***trnA*** | 4532-4597 | 66 | 27.3% |  |  | TGC |
| ***NCRI2*** | 4598-5329 | 732 | 30.7% |  |  |  |
| ***rrnS*** | 5330-6005 | 676 | 30.3% |  |  |  |
| ***cox2*** | 5999-6661 | 663 | 34.7% | ATA | TAG |  |
| ***trnS2 (UCN)*** | 6659-6722 | 64 | 29.7% |  |  | TGA |
| ***trnV*** | 6721-6783 | 63 | 36.5% |  |  | TAC |
| ***trnG*** | 6783-6842 | 60 | 16.7% |  |  | TCC |
| ***cox3*** | 6843-7625 | 783 | 31.9% | ATA | TAA |  |
| ***rrnL*** | 7626-8727 | 1102 | 27.7% |  |  |  |
| ***trnY*** | 8728-8788 | 61 | 31.1% |  |  | GTA |
| ***trnF*** | 8808-8865 | 58 | 43.1% |  |  | GAA |

^a^Underlined genes are on the minority strand. Genes not underlined are on the majority strand.
