# Supplementary material for: The Highly Divergent Mitochondrial Genomes Indicate That the Booklouse, Liposcelis bostrychophila (Psocoptera: Liposcelididae) Is a Cryptic Species
Source: G3 (Bethesda). 2018 Jan 19;8(3):1039–47. doi: 10.1534/g3.117.300410 (PMC5844292; doi:10.1534/g3.117.300410)
Supplement: Supplementary file 8 [file 1039TableS6.docx]

**Table S6.** Chromosome II of *Liposcelis bostrychophila* collected from Beijing(Group 1).

| **Gene^a^** | **Region** | **Size (bp)** | **GC%** | | **Start codon** | | **Stop codon** | | **Anticodon** |
| --- | --- | --- | --- | --- | --- | --- | --- | --- | --- |
| ***cox1*** | 1-1536 | 1536 | 35.0% | | ATG | | TAA | |  |
| ***trnD*** | 1528-1591 | 64 | 26.6% | |  | |  | | GTC |
| ***nad4L*** | 1593-1850 | 258 | 23.3% | | ATT | |  | |  |
| ***trnS1*** | 1856-1915 | 60 | 40.0% | |  | |  | | TCT |
| ***nad2*** | 1923-2774 | 852 | 28.3% | | ATT | | TAA | |  |
| ***trnT*** | 2830-2892 | 63 | 17.5% | |  | |  | | TGT |
| ***trnR*** | 2934-2985 | 52 | 40.4% | |  | |  | | TCG |
| ***NCRII1*** | 2986-3197 | 212 | 27.8% | |  | |  | |  |
| ***trnM*** | 3198-3257 | 60 | 28.3% | |  | |  | | CAT |
| ***trnE*** | 3278-3331 | 54 | 24.1% | |  | |  | | TTC |
| ***NCRII2*** | 3332-3411 | 80 | 23.8% | |  | |  | |  |
| ***trnW*** | 3412-3476 | 65 | 18.5% | |  | |  | | TCA |
| ***cob*** | 3511-4584 | 1074 | 33.8% | | ATT | | TAA | |  |
| ***nad6*** | 4581-5033 | 453 | 23.4% | | ATT | | TAA | |  |
| ***NCRII3*** | 5034-5157 | 124 | 33.1% | |  | |  | |  |
| ***trnL1 (CUN)*** | 5179-5247 | 69 | 23.2% | |  | |  | | TAG |
| ***trnI*** | 5243-5305 | 63 | 31.7% | |  | |  | | GCT |
| ***trnC*** | 5309-5370 | 62 | 33.9% | |  | |  | |  |
| ***IR*** | 5362-6242 | 881 | 29.9% | |  | |  | |  |
| ***p-atp6*** | 5363-5431 | 69 | 30.4% | |  | |  | |  |
| ***NCRII4*** | 5432-5558 | 127 | 29.1% | |  | |  | |  |
| ***trnA*** | 5559-5624 | 66 | 27.3% | |  | |  | | TGC |
| ***NCRII5*** | 5625-6243 | 619 | 30.2% | |  | |  | |  |
| ***p-nad5*** | 6244-6495 | 252 | 29.0% | |  | |  | |  |
| ***NCRII6*** | 6496-6644 | 149 | 33.6% | |  | |  | |  |
| ***trnK*** | 6645-6704 | 60 | 26.7% | |  | |  | | TTT |
| ***NCRII7*** | 6705-7154 | 450 | 36.2% | |  | |  | |  |
| ***p-nad1*** | 7155-7289 | 135 | 38.5% | |  | |  | |  |
| ***trnP*** | 7306-7370 | 65 | 18.5% | |  | |  | | TGG |
| ***trnQ*** | 7364-7426 | 63 | 25.4% | |  | |  | | TTG |
| ***nad3*** | 7424-7765 | 342 | 27.8% | | ATT | | TAG | |  |
| ***trnL2 (UUR)*** | 7767-7825 | 59 | 33.9% | |  | |  | | TAA |
| ***trnK*** | 7872-7939 | 68 | 16.2% | |  | |  | | CTT |
| ***NCRII8*** | 7940-8029 | 90 | 27.8% | |  | |  | |  |
| ***p-nad4*** | 8030-8206 | 177 | 0 | 22.6% | |  | |  | |
| ***p-nad1*** | 8207-8541 | 335 | 5 | 31.9% | |  | |  | |

^a^Underlined genes are on the minority strand. Genes not underlined are on the majority strand.
