# Supplementary material for: The Highly Divergent Mitochondrial Genomes Indicate That the Booklouse, Liposcelis bostrychophila (Psocoptera: Liposcelididae) Is a Cryptic Species
Source: G3 (Bethesda). 2018 Jan 19;8(3):1039–47. doi: 10.1534/g3.117.300410 (PMC5844292; doi:10.1534/g3.117.300410)
Supplement: Supplementary file 9 [file 1039TableS7.docx]

**Table S7.** Chromosome I of *Liposcelis bostrychophila* collected from Huangliangmeng (Group 2).

| **Gene^a^** | **Region** | **Size (bp)** | **GC%** | **Start codon** | **Stop codon** | **Anticodon** |
| --- | --- | --- | --- | --- | --- | --- |
| ***nad5*** | 1-1566 | 1566 | 31.4**%** | ATA | TAA |  |
| ***nad4*** | 1566-2774 | 1209 | 30.1**%** | ATC | TAA |  |
| ***nad1*** | 2774-3647 | 874 | 30.1% | ATC | T |  |
| ***atp8*** | 3648-3803 | 156 | 33.1% | GTG | TAG |  |
| ***atp6*** | 3769-4404 | 636 | 30.3% | ATA | TAA |  |
| ***IR*** | 4466-5451 | 986 | 29.8% |  |  |  |
| ***NCRI1*** | 4486-4594 | 109 | 33.0% |  |  |  |
| ***trnA*** | 4595-4658 | 64 | 25.0% |  |  | TGC |
| ***NCRI2*** | 4659-5143 | 485 | 29.5% |  |  |  |
| ***trnE*** | 5144-5200 | 57 | 24.6% |  |  | TTC |
| ***trnM*** | 5197-5255 | 59 | 28.8% |  |  | CAT |
| ***NCRI3*** | 5256-5459 | 204 | 24.5% |  |  |  |
| ***rrnS*** | 5460-6132 | 673 | 29.3% |  |  |  |
| ***cox2*** | 6132-6797 | 665 | 32.0% | ATA | TAA |  |
| ***trnS2*** | 6787-6849 | 63 | 31.7% |  |  | TGA |
| ***trnV*** | 6847-6911 | 65 | 30.8% |  |  | TCA |
| ***trnG*** | 6912-6972 | 61 | 23.0% |  |  | TCC |
| ***cox3*** | 6973-7755 | 783 | 33.0% | ATA | TAA |  |
| ***rrnL*** | 7756-8852 | 1097 | 36.4% |  |  |  |
| ***trnY*** | 8842-8902 | 61 | 31.1% |  |  | GTA |
| ***trnF*** | 8910-8982 | 73 | 27.4% |  |  | GAA |

^a^Underlined genes are on the minority strand. Genes not underlined are on the majority strand.
