# Supplementary material for: The Highly Divergent Mitochondrial Genomes Indicate That the Booklouse, Liposcelis bostrychophila (Psocoptera: Liposcelididae) Is a Cryptic Species
Source: G3 (Bethesda). 2018 Jan 19;8(3):1039–47. doi: 10.1534/g3.117.300410 (PMC5844292; doi:10.1534/g3.117.300410)
Supplement: Supplementary file 10 [file 1039TableS8.docx]

Table S8. Chromosome II of *Liposcelis bostrychophila* collected from Huangliangmeng (Group 2).

| **Gene^a^** | **Region** | **Size (bp)** | **GC%** | **Start codon** | **Stop codon** | **Anticodon** |
| --- | --- | --- | --- | --- | --- | --- |
| ***cox1*** | 1-1542 | 1542 | 34.8% | ATT | TAA |  |
| ***trnD*** | 1535-1594 | 60 | 21.7% |  |  | GTC |
| ***nad4L*** | 1597-1842 | 246 | 23.2% | ATT | TAA |  |
| ***trnS1*** | 1865-1921 | 57 | 43.9% |  |  | TCT |
| ***nad2*** | 1903-2781 | 852 | 26.4% | ATT | TAA |  |
| ***NCRII1*** | 2782-2838 | 57 | 17.5% |  |  |  |
| ***trnT*** | 2839-2901 | 63 | 17.5% |  |  | TGT |
| ***trnR*** | 2919-2969 | 51 | 33.3% |  |  | TCG |
| ***NCRII2*** | 2970-3053 | 84 | 29.8% |  |  |  |
| ***pnad4*** | 3054-3122 | 69 | 29.0% |  |  |  |
| ***NCRII3*** | 3123-3333 | 211 | 28.0% |  |  |  |
| ***trnW*** | 3334-3395 | 62 | 16.1% |  |  | TCA |
| ***cob*** | 3407-4459 | 1053 | 32.9% | ATT | TAA |  |
| ***nad6*** | 4456-4905 | 450 | 27.1% | ATT | TAG |  |
| ***pnad5*** | 4927-5144 | 218 | 41.3% |  |  |  |
| ***trnL1*** | 5161-5222 | 62 | 29.0% |  |  | TAG |
| ***trnI*** | 5219-5282 | 64 | 34.4% |  |  | GAT |
| ***trnC*** | 5293-5348 | 56 | 39.3% |  |  | GCA |
| ***IR*** | 5345-6330 | 986 | 29.8% |  |  |  |
| ***NCRII4*** | 5349-5473 | 125 | 33.6% |  |  |  |
| ***trnA*** | 5474-5537 | 64 | 25.0% |  |  | TGC |
| ***NCRII5*** | 5538-6023 | 486 | 31.9% |  |  |  |
| ***trnE*** | 6024-6078 | 55 | 25.5% |  |  | TTC |
| ***trnM*** | 6076-6135 | 60 | 28.3% |  |  | CAT |
| ***NCRII6*** | 6136-6289 | 154 | 27.9% |  |  | TTT |
| ***trnK*** | 6290-6350 | 62 | 24.2% |  |  |  |
| ***NCRII7*** | 6352-6532 | 181 | 30.9% |  |  |  |
| ***trnP*** | 6533-6591 | 59 | 20.3% |  |  | TGG |
| ***trnQ*** | 6587-6648 | 62 | 24.2% |  |  | TTG |
| ***nad3*** | 6649-6990 | 342 | 29.8% | ATA | TAG |  |
| ***trnL2*** | 6991-7051 | 61 | 27.9% |  |  | TAG |
| ***pnad5*** | 7074-7472 | 399 | 30.6% |  |  |  |
| ***NCRII8*** | 7473-7638 | 166 | 27.7% |  |  |  |

^a^Underlined genes are on the minority strand. Genes not underlined are on the majority strand.
