# Supplementary material for: The Highly Divergent Mitochondrial Genomes Indicate That the Booklouse, Liposcelis bostrychophila (Psocoptera: Liposcelididae) Is a Cryptic Species
Source: G3 (Bethesda). 2018 Jan 19;8(3):1039–47. doi: 10.1534/g3.117.300410 (PMC5844292; doi:10.1534/g3.117.300410)
Supplement: Supplementary file 11 [file 1039TableS9.docx]

**Table S9.** Chromosome I of *Liposcelis bostrychophila* collected from Kansas(Group 3).

| **Gene^a^** | **Region** | **Size (bp)** | **GC%** | **Start codon** | **Stop codon** | **Anticodon** |
| --- | --- | --- | --- | --- | --- | --- |
| ***rrnL*** | 1-1084 | 1,084 | 28.1% |  |  |  |
| ***pcox3*** | 1085-1406 | 322 | 32.3% |  |  |  |
| ***NCR5*** | 1407-1479 | 73 | 19.2% |  |  |  |
| ***pnad5*** | 1480-1587 | 108 | 25% |  |  |  |
| ***trnL2(TAA)*** | 1628-1688 | 61 | 26.2% |  |  |  |
| ***nad3*** | 1689-2030 | 342 | 29.2% | ATG | TAG |  |
| ***trnQ*** | 2030-2093 | 64 | 28.1% |  |  | TTG |
| ***trnP*** | 2089-2147 | 59 | 23.7% |  |  | TGG |
| ***NCRII4*** | 2148-2344 | 197 | 37.6% |  |  |  |
| ***trnK*** | 2345-2407 | 63 | 31.7% |  |  | TTT |
| ***IR*** | 2359-3194 | 836 | 30% |  |  |  |
| ***NCRII3*** | 2408-2514 | 107 | 27.1% |  |  |  |
| ***trnM*** | 2515-2573 | 59 | 28.8% |  |  | CAT |
| ***trnE*** | 2571-2626 | 56 | 17.9% |  |  | TTC |
| ***NCRII2*** | 2627-3110 | 484 | 34.1% |  |  |  |
| ***trnA*** | 3111-3176 | 66 | 22.7% |  |  | TGC |
| ***NCRII1*** | 3177-3194 | 18 | 16.7% |  |  |  |
| ***IR*** | 3195-3304 | 110 | 36.4% |  |  |  |
| ***NCRII1*** | 3195-3380 | 186 | 33.9% |  |  |  |
| ***atp6*** | 3381-4016 | 636 | 32.9% | ATA | TAA |  |
| ***atp8*** | 3982-4138 | 157 | 31.8% | TGT | TAA |  |
| ***nad1*** | 4138-5011 | 874 | 32.5% | ATC | T |  |
| ***nad4*** | 5011-6219 | 1,209 | 29.9% | ATC | TAA |  |
| ***nad5*** | 6220-7788 | 1,569 | 30.8% | ATT | TAG |  |
| ***trnF(GAA)*** | 7803-7872 | 70 | 27.1% |  |  | GAA |
| ***trnY(GTA)*** | 7872-7932 | 61 | 37.7% |  |  | GTA |

^a^Underlined genes are on the minority strand. Genes not underlined are on the majority strand.
