# Supplementary material for: The Highly Divergent Mitochondrial Genomes Indicate That the Booklouse, Liposcelis bostrychophila (Psocoptera: Liposcelididae) Is a Cryptic Species
Source: G3 (Bethesda). 2018 Jan 19;8(3):1039–47. doi: 10.1534/g3.117.300410 (PMC5844292; doi:10.1534/g3.117.300410)
Supplement: Supplementary file 12 [file 1039TableS10.docx]

**Table S10.** Chromosome II of *Liposcelis bostrychophila* collected from Kansas(Group 3).

| **Gene^a^** | **Region** | **Size (bp)** | **GC%** | **Start codon** | **Stop codon** | **Anticodon** |
| --- | --- | --- | --- | --- | --- | --- |
| ***cox1*** | 1-1542 | 1542 | 37.0% | ATC | TAA |  |
| ***trnD*** | 1535-1595 | 61 | 21.3% |  |  | GTC |
| ***nad4L*** | 1598-1843 | 246 | 26.0% | ATT | TAA |  |
| ***trnS1*** | 1864-1922 | 59 | 14.3% |  |  | TCT |
| ***nad2*** | 1930-2781 | 852 | 27.3% | ATT | TAA |  |
| ***NCRI1*** | 2782-2836 | 55 | 25.5% |  |  |  |
| ***trnT*** | 2837-2898 | 62 | 19.4% |  |  | TGT |
| ***trnR*** | 2916-2966 | 51 | 37.3% |  |  | TCG |
| ***NCRI2*** | 2967-3018 | 52 | 38.5% |  |  |  |
| ***P-nad4*** | 3019-3234 | 216 | 25.9% |  |  |  |
| ***trnW*** | 3269-3330 | 62 | 17.7% |  |  | TCA |
| ***cob*** | 3345-4397 | 1045 | 34.0% | ATT | TAA |  |
| ***nad6*** | 4394-4840 | 447 | 27.5% | ATT | TAA |  |
| ***pnad5*** | 4855-5062 | 208 | 38.9% |  |  |  |
| ***trnL1*** | 5082-5142 | 61 | 23.0% |  |  | TAG |
| ***trnI*** | 5139-5202 | 64 | 28.1% |  |  | GAT |
| ***trnC*** | 5214-5270 | 57 | 36.8% |  |  | GCA |
| ***IR*** | 5274-6219 | 946 | 30.8% |  |  |  |
| ***NCRI3*** | 5271-5402 | 132 | 32.6% |  |  |  |
| ***trnA*** | 5403-5466 | 64 | 23.4% |  |  | TGC |
| ***NCRI4*** | 5467-5952 | 486 | 34.0% |  |  |  |
| ***trnE*** | 5953-6006 | 54 | 18.5% |  |  | TTC |
| ***trnM*** | 6005-6063 | 59 | 28.8% |  |  |  |
| ***NCRI5*** | 6064-6168 | 105 | 27.6% |  |  |  |
| ***rrnS*** | 6220-6893 | 674 | 32.2% |  |  |  |
| ***cox2*** | 6894-7550 | 657 | 33.9% | ATA | TAA |  |
| ***trnS2*** | 7554-7618 | 65 | 33.8% |  |  | TGA |
| ***trnV*** | 7616-7679 | 64 | 27.1% |  |  | TAC |
| ***trnG*** | 7681-7741 | 61 | 19.7% |  |  | TCC |
| ***cox3*** | 7742-8524 | 783 | 33.7% | ATA | TAA |  |

^a^Underlined genes are on the minority strand. Genes not underlined are on the majority strand.
